# A comparison of the quality assurance of four dosimetric tools for intensity modulated radiation therapy

**DOI:** 10.1515/raon-2015-0021

**Published:** 2015-08-21

**Authors:** Jaeman Son, Taesung Baek, Boram Lee, Dongho Shin, Sung Yong Park, Jeonghoon Park, Young Kyung Lim, Se Byeong Lee, Jooyoung Kim, Myonggeun Yoon

**Affiliations:** 1 Department of Bio-Convergence Engineering, Korea University, Seoul, Korea; 2 Proton Therapy Center, National Cancer Center, Goyang, Korea; 3 McLaren Proton Therapy Center, Karmanos Cancer Institute, Flint, MI, USA; 4 Department of Radiation Oncology, Ilsan Hospital, Goyang, Korea; 5 Department of Radiation Oncology, Sun Hospital, Daejeon, Korea

**Keywords:** intensity modulated radiation therapy, quality assurance, dosimetric tool, gamma index

## Abstract

**Background:**

This study was designed to compare the quality assurance (QA) results of four dosimetric tools used for intensity modulated radiation therapy (IMRT) and to suggest universal criteria for the passing rate in QA, irrespective of the dosimetric tool used.

**Materials and methods.:**

Thirty fields of IMRT plans from five patients were selected, followed by irradiation onto radiochromic film, a diode array (Mapcheck), an ion chamber array (MatriXX) and an electronic portal imaging device (EPID) for patient-specific QA. The measured doses from the four dosimetric tools were compared with the dose calculated by the treatment planning system. The passing rates of the four dosimetric tools were calculated using the gamma index method, using as criteria a dose difference of 3% and a distance-to-agreement of 3 mm.

**Results:**

The QA results based on Mapcheck, MatriXX and EPID showed good agreement, with average passing rates of 99.61%, 99.04% and 99.29%, respectively. However, the average passing rate based on film measurement was significantly lower, 95.88%. The average uncertainty (1 standard deviation) of passing rates for 6 intensity modulated fields was around 0.31 for film measurement, larger than those of the other three dosimetric tools.

**Conclusions:**

QA results and consistencies depend on the choice of dosimetric tool. Universal passing rates should depend on the normalization or inter-comparisons of dosimetric tools if more than one dosimetric tool is used for patient specific QA.

## Introduction

Radiation therapy is one of the most widely used cancer treatment methods. Among the methods used are 3D conformal radiation therapy (3D CRT), intensity modulated radiotherapy (IMRT), and particle beam therapy. IMRT uses a multi-leaf collimator (MLC) to vary the intensity of the beam delivered to the tumour.^1^ Since IMRT requires fine control of the MLC during irradiation, caution must be exercised in delivering radiation.^2^–^4^ Treatment quality assurance (QA) is therefore necessary to determine the difference between calculated and actual dose distributions.^5^,^6^

QA for conventional IMRT treatment is widely performed using an ion chamber and film, with the ion chamber used to measure absolute dose at each location and film used for 2D relative dose comparisons.^7^–^9^ Although film has the great advantage of high resolution, it has several disadvantages, including the need to change film for every beam test and its dependence on beam energy, processing conditions, and external light. Recently developed radiation therapy methods have greater accuracy, with new dosimetry tools developed to overcome the disadvantages of film measurements. Among these tools are a diode detector-based 2D diode array dosimeter^10^ (MapCheck2; Sun Nuclear Corporation, Melbourne, Florida), a 2D ion chamber array dosimeter^11^ (I’mRT MatriXX; IBA) and portal dosimetry using an electronic portal imaging device^12^ (EPID). The gamma evaluation method is generally used to verify the actual dose distribution that will be delivered to the patient during IMRT.^13^ This method is based on a comparison of the calculated 2D dose map from treatment planning system (TPS) with the measured 2D dose map from each dosimetric tool. Although there is no general consensus, QA results are considered acceptable when the passing rate is greater than 95% using as criteria a tolerance of dose difference (DD) of 3% and a tolerance for distance to agreement (DTA) of 3 mm.^14^

Table 1 shows an example of QA results using film, Mapcheck, MatriXX and portal dosimetry based on gamma evaluation for 10 randomly selected treatment plans undergoing IMRT at four different institutions in Korea. Each of 4 hospitals sorted out 10 IMRT QA results using one of four dosimetric tools and analysed the passing rates depending on the QA tools. Therefore, this data shows the general passing rates of IMRT QA depending on the dosimetric tools. The mean ± standard deviation (SD) passing rates (γ% ≤ 1) for film, Mapcheck, MatriXX and EPID were 96.80 ± 0.94%, 98.90 ± 0.55%, 99.40 ± 0.48% and 97.10 ± 1.12%, respectively. Thus, passing rates are dependent on the dosimetric tool used, with mean passing rates being lowest for film and highest for MatriXX. This example suggests that passing rates of 95% for film measurement and portal dosimetry are not equivalent.^15^,^16^ Many institutions, however, have not set an acceptance level for each tool but have universally set 95% as the acceptance level for all tools. The current emphasis on treatment QA for patients suggests the need for specific guidelines for each specific dosimetric tool. Although guidelines have been proposed^17^, they were only for acceptable doses and allowable errors for each body part, and did not include passing rates for different dosimetric tools.

In this study, four different dosimetric tools were used for quality assurance method of IMRT plans from five patients and their patient specific QA results were compared. The correlations among these dosimetric tools were used to determine reasonable tolerance levels for each.

## Materials and methods

**FIGURE 1.**
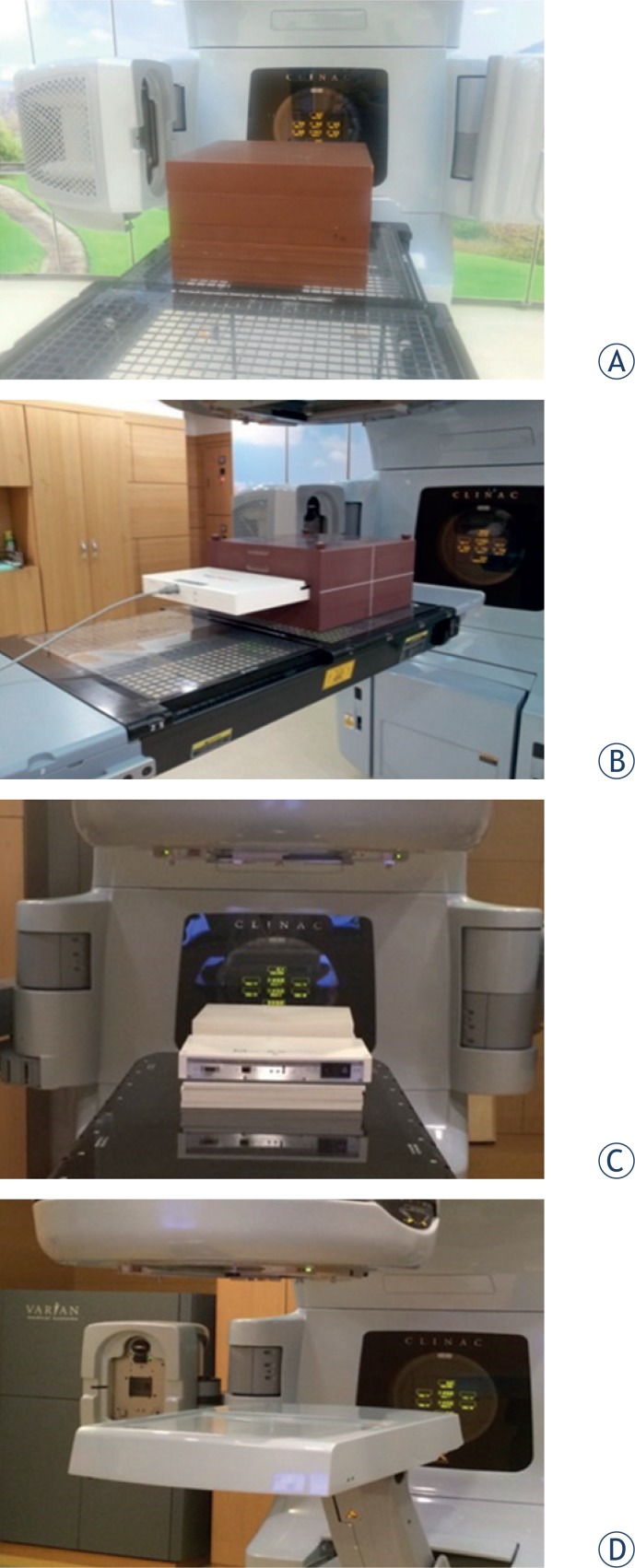
Pictures of the experimental setup for IMRT QA using various dosimetric tools. **(A)** Film, **(B)** Mapcheck, **(C)** ion chamber array (MatriXX), **(D)** Portal dosimetry.

The IMRT plans for five patients undergoing radiation therapy, modelled using the Eclipse treatment planning system Ver. 8.9. (Varian Medical Systems, Salt Lake City, UT, USA) with anisotropic analytical algorithm (AAA), were used for this study.^18^ Figure 1 shows the experimental setup of IMRT QA with film, Mapcheck, MatriXX and EPID. The gamma index method was used to compare the TPS dose with measured doses, using dose difference (DD) criteria of 3% and distance-to-agreement criteria (DTA) of 3 mm. The reference dose map was the calculated dose maps from TPS in our analysis. The gamma index value was calculated at all 2-dimensional points, with the percentage of points with a gamma index value ≤ 1 and meeting the DD and DTA criteria being the passing rate. The passing rates of the four dosimetric tools were compared.

### Film QA

The traditional method of QA for IMRT is 2-dimensional testing using film. We used commercial Gafchromic EBT2 film (International Specialty Products, New Jersey, USA)^19^ and an Epson Expression 10000-XL flatbed film scanner (Epson, California, USA), with a resolution of 0.38 × 0.38 mm^2^. Film was calibrated using an ion chamber, with more calibration points used for low dose areas to enhance accuracy. Doses were measured at a source to axis distance (SAD) of 100 cm, with film located at a depth of 10 cm of solid slab phantom and a gantry angle of 0°. Radiological Imaging Technology IMRT software (Ver. 5.2, Colorado Springs, CO, USA) was used to verify dose delivery after the beam measurement.

### MapCheck QA

MapCheck2 (Sun Nuclear; Melbourne, FL, USA) is a relatively new dosimetric tool, consisting of diode detectors in a 2D array and a field size of 32 cm × 26 cm. The matrix is composed of 1,527 diodes, spaced 7.07 mm apart, with each having an active detector area of 0.64 mm^2^ and the entire matrix having active detector size of 24.4 × 24.4 cm^2^.^20^ Similar to film QA, dose was measured at 8 cm depth of Mapcheck dedicated phantom, Mapphan (Sun Nuclear, Melbourne, FL, USA) at a gantry angle of 0°. Mapcheck dedicated software (MapCHECK2, Ver 5.01.05, Sun Nuclear, Melbourne, FL, USA), was used to verify the dose delivery after the beam measurement.

### MatriXX QA

The MatriXX (IBA Dosimetry GmbH, Schwarzenbruck, Germany) is similar to Mapcheck but has ionization chambers rather than diode detectors in a 2D array.^21^,^22^ Although MatriXX has fewer ion chambers than Mapcheck has diode detectors, the ion chambers yield more stable data than the diodes.^23^ The 1,024 ionization chambers of MatriXX are aligned in a parallel pattern in a 32 × 32 grid, with the diameter, height, volume and detector spacing of each ion chamber being 4.5 mm, 7.62 mm, 0.08 cc and 7.62 mm, respectively. The gantry angle was 0° and the beam was investigated using a 5 cm solid water phantom on top of the MatriXX. OmniPro-I’mRT (Ver 1.7.0021, IBA Dosimetry, Germany), a MatriXX-dedicated software program, was used to verify dose delivery after beam irradiation.

### EPID QA (portal dosimetry)

We used an aSi-based EPID (aS500, Varian Medical Systems, Palo Alto, CA) attached to a Varian Clinac iX Linear accelerator (Varian Medical Systems, Palo Alto, CA, USA).^12^ This EPID has a resolution of 0.784 × 0.784 mm^2^ with an array of 512 × 384 pixels, thus having higher resolution than Mapcheck or MatriXX, and a field size of 40 × 30 cm^2^. The source to axis distance (SAD) was set at 100 cm and the beam was investigated at a gantry angle of 0°. Measurement with EPID was measured with no phantom and EPID dedicated software (Eclipse, Ver 8.9, Varian Medical System, USA) was used to verify dose delivery after the beam measurement.

## Results

**FIGURE 2.**
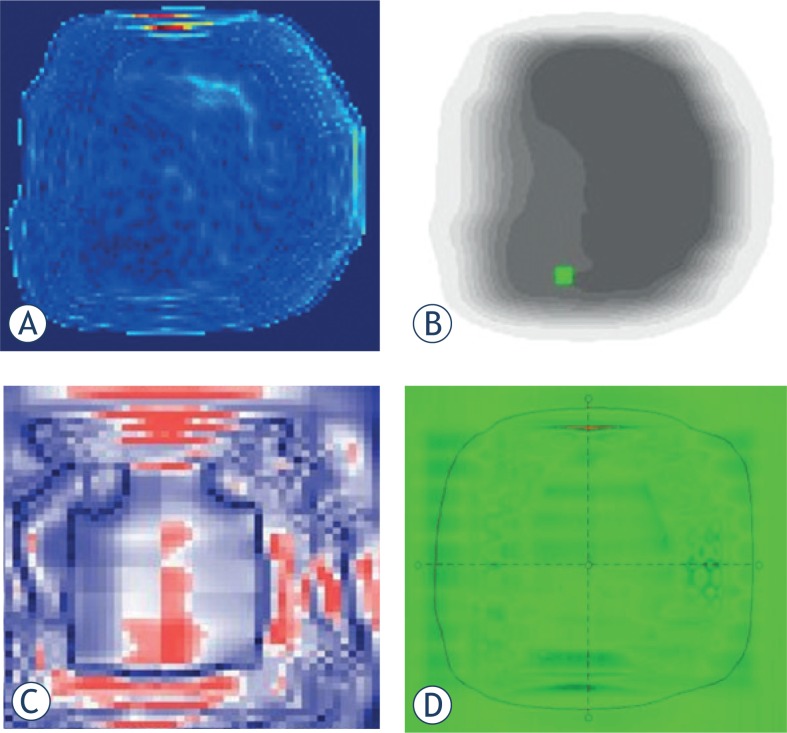
2D images of the passing rate based on gamma evaluation for various dosimetric tools. **(A)** Film, **(B)** diode array (Mapcheck), **(C)** ion chamber array (MatriXX), **(D)** Portal dosimetry.

Doses calculated using TPS were compared with doses measured by the four dosimetric tools based on gamma evaluation (3%/3mm, threshold 15%). Figure 2 shows examples of gamma evaluation results using film, Mapcheck, MatriXX and EPID for IMRT QA. Although Table 1 shows the general passing rates of IMRT QA depending on the dosimetric tools, the result can be dependent on the patient selected in each institution. To clarify the dependency of QA result on dosimetric tools, one should carry out IMRT QA of *same* patient using *different* dosimetric tools. Table 2 shows the mean passing rates, based on the gamma index method, for the treatment fields of each patient using film, Mapcheck, MatriXX, and EPID. The values measured with the four dosimetry tools showed good agreement with the calculated values for all five patients. The mean ± standard deviation (SD) passing rates (γ% ≤ 1) for film, Mapcheck, MatriXX and EPID for 30 IMRT fields of five patients were 95.88 ± 0.87%, 99.61 ± 0.41%, 99.04 ± 0.18% and 99.29 ± 0.15%, respectively. Although all four dosimetry tools met the acceptable passing rate of 95%, these tools showed some differences in measuring the same beam, with the gamma index being much lower for film than for the three other tools.

To show fluctuations for each dosimetric tool, we assessed the passing rates of three consecutive measurement results using film, Mapcheck, MatriXX and EPID based on gamma index values for 6 fields of patient 1 from Table 2. This means that QA was carried out 3 times, repetitively. As shown in Table 3, the gamma index results were similar, regardless of the number of measurements, for Mapcheck, MatriXX and EPID. Film, however, showed higher a standard deviation (i.e., fluctuation) for three consecutive measurements than for the other dosimetirc tools. For example, the fluctuation of film measurement for field 1 was 0.59 which is much higher than 0.00, 0.00, 0.05 for Mapcheck, MatriXX and EPID, respectively.

## Discussion

This comparison of gamma indices for EBT film, Mapcheck, MatriXX, and EPID showed differences in dose distribution when using these various dosimetric tools to carry out the quality assurance for the same patients undergoing IMRT. Even with using the same dosimetry tool, the results differed slightly for each measurement of the same field.

The passing rates based on film measurement were much lower than those using the three other dosimetric tools (Table 2). To check the uncertainty of film for the exact same beam, QA was carried out *only one time* but *3 films* were inserted in the phantom. To irradiate the exactly same beam, one should remove the unstable low dose part of the beam caused by scattering, leakage, etc. To do that, we did carry out QA *not with individual field* but *with composite fields,* which may remove the low dose part in the analysis*.* Assessment of the passing rates based on gamma evaluation showed uncertainties ranging from 0.11 to 0.39 (Table 4). Despite these uncertainties, however, the results were reasonably stable, suggesting that a single measurement would be sufficient for QA.

In general, uncertainties tend to be higher for low dose film measurements.^20^ We therefore investigated a beam with a universally-tripled-beam intensity (i.e., tripled monitor unit) on each field to confirm the decrease of gamma index with low dose. Table 5 shows the passing rate using film measurements for 3-fold increased beam intensity for 6 fields of patient 1, 2, 4. Compared with the results shown in Table 3, the mean passing rate increased, indicating that, in general, high and stable gamma index results with film can be obtained at higher beam intensity (i.e., increased monitor units), comparable to the results from the three other dosimetric tools. Table 5 also shows that standard deviation is decreased with increased beam intensity. Therefore, QA using film should be performed at a sufficiently high intensity beam to reduce the uncertainties from low doses. Although raising the threshold level will lead to clearing of low dose, it may not appropriate since it can cause only the high dose area to be set as region of interest. If one wants to use normal treatment MU in QA, careful calibration of film should be used to decrease the standard deviation of film for low dose area.

While the QA results based on Mapcheck, MatriXX and EPID showed good agreement among themselves by showing average passing rates of 99.61%, 99.04% and 99.29%, respectively, the average passing rate based on film measurement was significantly lower, 95.88%. The QA result seems to depend on the unique characteristics of each dosimetric tool such as depth of measurement, its resolution, etc. These experimental results suggest that the criteria of the passing rate based on film measurement should be decreased about 3% compared to the criteria for other dosimetric tools. If one wants to use universal criteria for the passing rate for all four dosimetric tools, one should increase the beam intensity about three times if the film is used as a measurement tool. Further study is needed to suggest the more reliable universal passing rate with large number of case studies.

## Conclusions

We have used film, Mapcheck, MatriXX and EPID to evaluate their dosimetric properties for IMRT QA. While the measured dose maps with Mapcheck, MatriXX and EPID agreed well with the calculated dose maps of TPS, the passing rate was noticeably lower for film measurements. It seems that different passing rates in QA results may partially stem from the different resolution (or measured dose matrix) of four dosimetric tools. Use of film for IMRT QA should be implemented using beams of sufficiently high intensity to be compatible with the results of Mapcheck, MatriXX, and EPID. Our results suggest that setting an acceptance level based on the correlation of various dosimetric tools is a more correct method than simply setting the same acceptance level for all of these tools.

## Figures and Tables

**TABLE 1.** Average passing rates for film, diode array (Mapcheck), ion chamber array (MatriXX) and electronic portal imaging device (EPID) for intensity modulated radiation therapy (IMRT) quality assurance (QA) in four different institutions in Korea

	**Film**	**Mapcheck**	**MatriXX**	**EPID**
**Mean**	96.80	98.90	99.40	97.10
**Standard deviation**	0.94	0.55	0.48	1.12

**TABLE 2.** Mean passing rates based on the gamma index method for the treatment fields of each patient using film, diode array (Mapcheck), ion chamber array (MatriXX), and electronic portal imaging device (EPID)

	**Patient 1**	**Patient 2**	**Patient 3**	**Patient 4**	**Patient 5**	**Mean**
**Film**	97.42	95.42	95.83	94.80	95.92	95.88
**Mapcheck**	100.00	99.45	100.00	98.90	99.70	99.61
**MatriXX**	99.10	99.26	98.84	98.82	99.20	99.04
**EPID**	99.42	99.12	99.20	99.52	99.20	99.29

**TABLE 3.** Passing rates of three consecutive measurement results using film, diode array (Mapcheck), ion chamber array (MatriXX) and electronic portal imaging device (EPID) based on gamma index values for 6 fields of patient 1. The data shown for patient 1 in Table 2 is the average of first measurement set of data in Table 3

	**Measurement**	**Field 1**	**Field 2**	**Field 3**	**Field 4**	**Field 5**	**Field 6**
**Film**	1^st^	98.95	98.97	91.20	97.86	98.88	98.66
	2^nd^	97.90	98.93	91.18	98.74	98.28	98.71
	3^rd^	97.58	98.42	91.15	99.24	98.65	98.26
	Mean (SD)	98.14 (0.59)	98.77 (0.25)	91.18 (0.02)	98.61 (0.57)	98.60 (0.25)	98.54 (0.20)
**Mapcheck**	1^st^	100.00	100.00	100.00	100.00	100.00	100.00
	2^nd^	100.00	100.00	100.00	100.00	100.00	100.00
	3^rd^	100.00	100.00	100.00	100.00	100.00	100.00
	Mean (SD)	100.00 (0.00)	100.00 (0.00)	100.00 (0.00)	100.00 (0.00)	100.00 (0.00)	100.00 (0.00)
**MatriXX**	1^st^	99.29	98.41	99.57	99.30	99.10	98.97
	2^nd^	99.30	99.25	99.57	99.28	99.06	99.01
	3^rd^	99.29	99.24	99.58	99.29	99.00	98.99
	Mean (SD)	99.29 (0.00)	98.97 (0.39)	99.57 (0.00)	99.29 (0.01)	99.05 (0.04)	98.99 (0.02)
**EPID**	1^st^	99.80	99.40	99.60	98.90	98.80	100.00
	2^nd^	99.90	99.60	99.70	99.20	99.10	100.00
	3^rd^	99.80	99.60	99.45	99.05	98.90	99.90
	Mean (SD)	99.83 (0.05)	99.53 (0.09)	99.58 (0.10)	99.05 (0.12)	98.93 (0.12)	99.97 (0.05)

SD = standard deviation

**TABLE 4.** Passing rates based on gamma evaluation using 3 films in the same location for intensity modulated radiation therapy (IMRT) quality assurance (QA) of each patient

	**Patient 1**	**Patient 2**	**Patient 3**	**Patient 4**	**Patient 5**
**Film**	98.36	95.29	98.51	98.08	99.41
	97.47	95.47	98.55	98.19	99.14
	97.63	95.65	98.87	98.36	99.27
**Mean**	97.82	95.47	98.64	98.21	99.27
**SD**	0.39	0.15	0.16	0.12	0.11

**TABLE 5.** Passing rates of film measurements based on gamma evaluation for 3-fold increased beam intensity (i.e., monitor unit) for 6 fields of patient 1

	**Field1**	**Field2**	**Field3**	**Field4**	**Field5**	**Field6**	**Mean**	**SD**
**Patient1**	99.23	99.45	99.20	99.09	99.04	99.21	99.20	0.13
**Patient2**	98.63	98.04	98.03	97.91	96.80	97.15	97.76	0.61
**Patient4**	98.77	98.67	99.23	99.40	99.93	98.30	99.05	0.53
